# Pressure-Driven Membrane Processes for Removing Microplastics

**DOI:** 10.3390/membranes15030081

**Published:** 2025-03-05

**Authors:** Priscila Edinger Pinto, Alexandre Giacobbo, Gabriel Maciel de Almeida, Marco Antônio Siqueira Rodrigues, Andréa Moura Bernardes

**Affiliations:** 1 Post-Graduation Program in Mining, Metallurgical and Materials Engineering (PPGE3M), Federal University of Rio Grande do Sul (UFRGS), Av. Bento Gonçalves, n. 9500, Porto Alegre 91509-900, RS, Brazil; gabrielmacielrs@gmail.com (G.M.d.A.); amb@ufrgs.br (A.M.B.); 2 Post-Graduation Program in Materials Technology and Industrial Processes, Feevale University, Rodovia RS-239, n. 2755, Vila Nova, Novo Hamburgo 93525-075, RS, Brazil; marcor@feevale.br

**Keywords:** microfiltration, microplastics, nanofiltration, nanoplastics, reverse osmosis, ultrafiltration

## Abstract

The intense consumption of polymeric materials combined with poor waste management results in the dissemination of their fragments in the environment as micro- and nanoplastics. They are easily dispersed in stormwater, wastewater, and landfill leachate and carried towards rivers, lakes, and oceans, causing their contamination. In aqueous matrices, the use of membrane separation processes has stood out for the efficiency of removing these particulate contaminants, achieving removals of up to 100%. For this review article, we researched the removal of microplastics and nanoplastics by membrane processes whose driving force is the pressure gradient. The analysis focuses on the challenges found in the operation of microfiltration, ultrafiltration, nanofiltration, and reverse-osmosis systems, as well as on the innovations applied to the membranes, with comparisons of treatment systems and the peculiarities of each system and each aqueous matrix. We also point out weaknesses and opportunities for future studies so that these techniques, known to be capable of removing many other contaminants of emerging concern, can subsequently be widely applied in the removal of micro- and nanoplastics.

## 1. Introduction

Produced mainly from the refining of oil and natural gas, polymers, popularly known as plastics, are present in in a large variety of items surrounding the human population. Their production process and properties justify their constant presence in the daily human routine. They are light, have electrical and thermal insulating characteristics, can be flexible, and have good optical properties [1]. Due to their widespread consumption, in 2019 annual global production reached 460 million tons, representing an increase of almost 230 times since the 1950s, when the rapid growth in plastic production began [2]. Furthermore, according to Ritchie et al. [3], approximately 0.5% of the global plastic waste generated is transported to the oceans, totaling somewhere between 1 and 2 million tons, representing alarming data regarding water pollution. Water is one of the most important routes by which microplastics are transferred from one place to another [4], which makes the presence of micro- and nanoparticles of plastics an emerging concern.

According to the vast majority of scientists in the field, microplastics (MPs) are defined as any polymeric particle smaller than 5 mm. Their origin may stem from the degradation of larger materials disposed of improperly, fragmenting in the oceans, or from weathering, as well as from fibers released during the washing of clothes and the production of synthetic fabrics. In all these cases, the particles are termed secondary MPs. In addition, a recent study [5] indicated that particles emitted by tire tread wear and asphalt pavement fragmentation residues represent a significant portion of secondary MPs in urban stormwater. On the other hand, MPs can be particles intentionally produced in this format, classified as primary MPs, which are present in personal care products such as toothpaste, soaps, and exfoliating creams [6].

In fact, the classification of MPs by size is already established, covering polymeric particles ranging from 1 μm to 5 mm [7,8,9]. However, particles smaller than 1 μm have also been detected and classified as nanoplastics (NPs) [10]. Since studies on NPs are incipient, their classification is still under development. Thus, the scientific literature typically classifies polymeric particles smaller than 1000 nm as NPs [9,10,11]; however, some studies consider only those smaller than 100 nm as NPs [12,13] while others consider particles between 1 and 1000 nm to be NPs [7,14]. These definitions can influence and shape directions in the elaboration of laws and standards, but in fact, regarding the environment, it is known that both particle sizes have harmful effects on the ecosystem. Furthermore, studies indicate that microplastics can act as bioaccumulators and adsorb heavy metals or other contaminants of emerging concern [15,16,17] increasing their toxicological potential. They can cause serious problems for aquatic fauna, as the ingestion of MPs can lead to gastrointestinal blockages and a feeling of satiety in animals without providing the necessary nutrients [17].

Similarly, there is already evidence of MPs in human organs, tissues, and fluids, including the lungs, brain, and placenta, and even in breast milk [18]. Although the effects of MPs in humans are still being studied, their ingestion or inhalation has already been associated with adverse effects in several organs. It is known that the presence of MPs measuring 44 to 60 µm in the intestine can alter the microbiota, causing dysfunction of the intestinal barrier, inflammation, and even cell death [19]. In organs such as the lungs, liver, and heart, in addition to the risks of inflammation and cell death, there are also potentially more severe effects. In the heart, MPs can impair the development of heart valves and form blood clots, resulting in cardiovascular problems [19]. Another recent highlight is the discovery of NPs and MPs in deep layers of the human brain: this condition is capable of causing an imbalance in energy production, reduced cell viability, and impaired development of brain organoids [20].

The problem related to the occurrence of MPs in humans is so great that the ingestion of MPs—which can be found in milk, honey, beer, and other edibles and potables—is equivalent to weekly values of 0.1 to 5 g, something that could be represented as the consumption of approximately one “credit card” per week [21]. This further highlights the need to include MP/NP removal methods in water and wastewater treatment systems [18,21,22]. Due to the prospects of increasing MPs in the ocean and the limited knowledge about the adverse effects they can cause on the environment [23], MPs can be considered contaminants of emerging concern [24]. Therefore, it is necessary to investigate and implement technologies capable of efficiently removing them.

**Figure 1:**
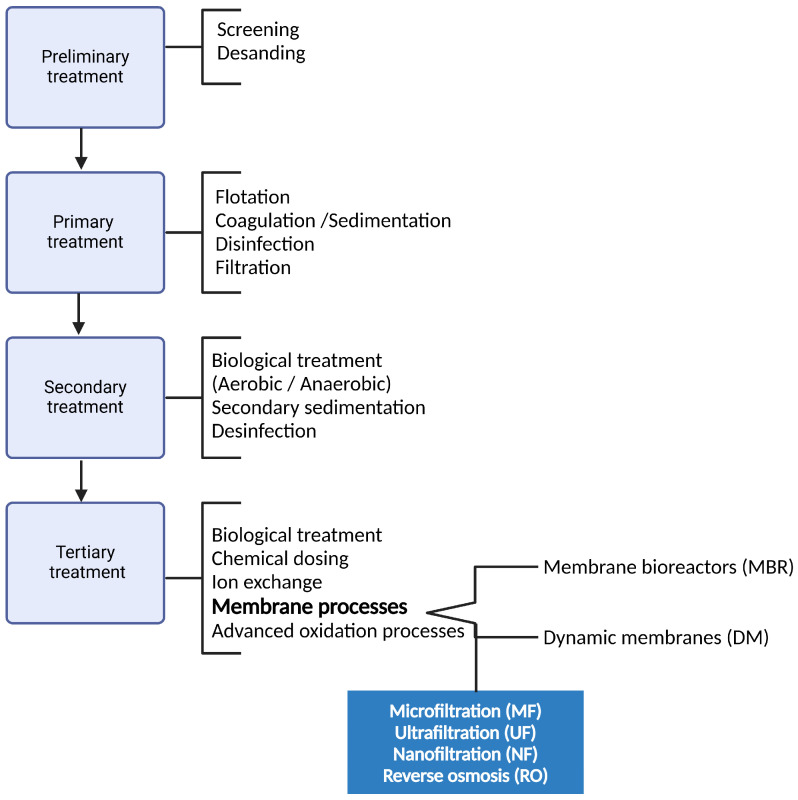
Stages and main processes used in wastewater treatment. Adapted from [25,30].

There are several studies proposing a variety of technologies for removing these contaminants from aqueous matrices. The most popular alternatives are physical treatment methods, such as adsorption, gravitational separation, conventional filtration, and membrane processes, as seen in Figure 1. Chemical (oxidation and coagulation, for example) and biological (activated sludge processes and anaerobic digestion) processes are also utilized, which can be combined in one or more treatment stages, such as primary, secondary, and tertiary [25,26]. Additionally, techniques such as dissolved air flotation [27] UV radiation, ozone [28] electrokinetic-assisted filtration [11] and even electrocoagulation, are being employed. Many of these methods, from the simplest to the most advanced, have the potential to remove MPs, but like membrane processes, they also present some drawbacks [28,29].

With this in mind, this review analyzes studies carried out on the removal of MPs using pressure-driven membrane processes, namely microfiltration (MF), ultrafiltration (UF), nanofiltration (NF), and reverse osmosis (RO). Several factors influence MP removal efficiency by membrane processes, such as pore size, membrane charge, dipole moment, and hydrophilicity [9]. Through the studies already published on the subject, these membrane processes will be assessed based on aspects such as difficulties, advantages, and opportunities, as well as suggestions for future work. Despite the distinction in the literature between MPs and NPs, throughout this paper we will use the generic term MPs, which encompasses plastic particles smaller than 5 mm. We will return to the term NPs only in specific cases where it is necessary to differentiate them from MPs.

## 2. Methodology

The data for this review were extracted from the Scopus database through searches performed on 23 December 2024 and 22 February 2025 using the following keywords: “microplastic” AND “removal” OR “catch” OR “rejection” OR “retention” AND “microfiltration” OR “ultrafiltration” OR “nanofiltration” OR “reverse osmosis”. The search was limited to research articles published from 2017 to 31 December 2024. Among the 107 articles found, those whose applied methodologies included the four pressure-driven membrane processes (PDMPs) for removing MPs were selected for data processing. For this reason, 44 articles were removed, since some did not encompass both subjects, MPs and PDMPs, used another type of membrane separation process, or were focused on MP quantification studies and did not add important information to the present study. Another 31 references were added due to a screening in the references of the selected articles or included to ground some information, totaling 94 references in this review. It is worth mentioning that MPs are a current topic, which justifies the absence of articles before 2017 in the search with the keywords we used. The term microplastic itself was incorporated into the scientific literature only in 2004 by Thompson et al. [31] and since then, countless studies have been developed in several areas of knowledge.

## 3. Removal of MPs from Aqueous Matrices by Pressure-Driven Membrane Processes

**Figure 2:**
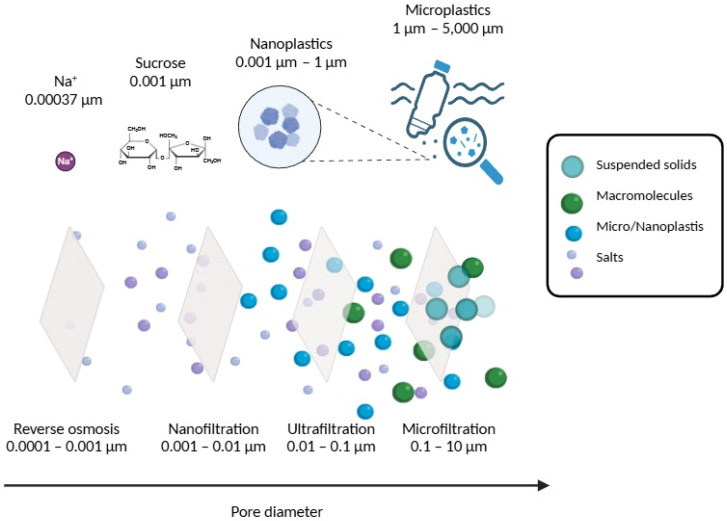
Pressure-driven membrane processes, range of membrane pore diameters, and some examples of contaminants capable of being removed.

Pressure-driven membrane processes (PDMPs) can segregate solid particles such as microplastics and nanoplastics from fluids depending on their size. Separation methods in which exclusion occurs by size, as represented in Figure 2, seem to be a good strategy, since the interactions of plastic particles with their surroundings encompass several phenomena, such as van der Waals forces, electrostatic repulsion, adsorption, steric interactions, hydrophobic interactions, size exclusion, and hydration forces [32,33]. For this reason, for adequate retention of MPs, it is necessary to pay attention to the properties of the solution, the hydrodynamic conditions, and other parameters that can influence the separation.

In PDMPs, the membrane acts as a selective barrier, allowing certain substances, such as molecules, ions, or small particles, to permeate, while blocking others. PDMPs are categorized based on membrane pore size for the purposes of microfiltration, ultrafiltration, nanofiltration, and reverse osmosis [34] Membranes are typically produced with organic such polymers as polytetrafluoroethylene (PTFE), polyethylene terephthalate (PET), polyvinylidene fluoride (PVDF), polypropylene (PP), polyethylene (PE), polyamide (PA), polysulfone (PSU), and polyethersulfone (PES) [35] as well as with biodegradable materials such as polylactic acid [36]. The use of polymeric membranes in separation processes is considered advantageous due to their characteristics, such as low energy consumption, absence or minimization of chemicals, ease in scaling up, and high efficiency even under mild operating conditions [37,38]. These characteristics benefit applications in drinking water treatment systems, wastewater, or other purposes, such as in the separation stage in producing agroindustrial products or other products for human consumption [39]. Research relating to the usage of PDMPs for MP removal is very diverse, with the vast majority of PDMPs showing excellent removal performance, whether using commercial membranes, modified membranes, or membranes produced in research laboratories [40,41,42].

### 3.1. Microfiltration

MF uses membranes with pore sizes ranging from 0.1 to 10 µm and operating pressures from 0.01 to 2 bar. It is usually applied to remove bacteria and suspended solids and to treat water and wastewater [43]. MF is sometimes employed as a pretreatment for operations with tighter membranes such as NF or RO, contributing to the reduction in fouling in the membranes of downstream processes in water treatment plants (WTPs) or wastewater treatment plants (WWTPs), as reported by Barbier et al. [8], whose research evaluated a WTP in Paris that had MF as a pretreatment for nanofiltration [8,44]. Because it uses larger pores and lower working pressure, which reduce its operating costs compared to other PDMPs, MF can be used in supplementary drinking water filtration devices for end consumers.

In a study conducted by Cherian et al. [23], MP removal capacity was assessed by comparing three different types of domestic filters combining MF, activated carbon, and ion exchange, one without a membrane, one using microfiltration, and the other with a geotextile membrane. The best performance was achieved using the device equipped with MF (pore opening ≥ 0.2 µm), capable of retaining 93.6% ± 2.2% of the polyvinyl chloride (PVC), polyethylene terephthalate (PET), and nylon microfiber particles. It should be noted that due to the impossibility of performing a backwash, the consumer must change the filters according to the manufacturer’s instructions in order to maintain their efficiency in removing microplastics and other contaminants [23].

In a study by Pizzichetti et al. [45], microfiltration membranes were used for polishing treated water. In the three membranes tested, polycarbonate (PC), cellulose acetate (CA), and polytetrafluoroethylene (PTFE), whose pore size was 5 µm, the microparticles of PA and polystyrene (PS) were efficiently removed. Table 1 shows the average particle sizes, as well as details on the operation of the membrane systems. Although all membranes removed >94% of MPs, reaching very close to 100% in PA removal, the study highlights some factors that may influence the permeation of microplastics through the membranes, such as low mechanical resistance of some membranes, irregular shape of some MPs, and MP fracture when subjected to the filtration stage. Other important factors are the abrasion of MPs on the membrane and hydrophobicity characteristics. The authors concluded that the most recommended membrane among those tested was the CA membrane, due to its performance in terms of MP removal, operating pressure, and flow pattern [45].

Hyeon et al. [46] tested two ceramic MF membranes, one made of alumina and the other of silicon carbide, to remove MPs from laundry wastewater. Both membranes demonstrated good performance in removing MPs, particularly the alumina membrane, which was attributed to its narrower pore size distribution. However, the silicon carbide membrane exhibited better performance in terms of permeate flux, with a smaller decline throughout the tests.

Kook et al. [47] evaluated the behavior of alumina ceramic membranes in removing PE microplastics in the presence of hydrophobic compounds used as organic UV filters, contaminants of emerging concern commonly found in personal care products designed to protect the skin against UV radiation. They observed the adsorption of the organic UV filters onto the PE microplastics through hydrophobic interactions, which increased the removal efficiency of the organic UV filters from 34.2–37.8% (without MPs) to 82.2–97.9% (with MPs), as the MPs were nearly completely removed.

In another study, Kook et al. [48] compared three MF membranes with a nominal pore size of 0.1 μm, two of which were polymeric (PVDF) and one ceramic (alumina), in removing PE and PS microplastics with sizes of 0.1 and 1 μm. The authors observed a sharp decline in flux during the first 15–20 minutes of experiments with 0.1 µm PS microplastics for all three membranes. Thereafter, it remained relatively constant at values below 10% of the initial flux. This significant loss of productivity was attributed to fouling in all three membranes caused by standard or complete pore blocking, since these MPs were 0.1 µm in size, equal to the average pore size of the membranes. Particles with sizes very close to the pore size can penetrate and attach to the inner walls of the membrane pores, leading to severe membrane fouling. The authors also emphasize that both polymeric and ceramic membranes can remove a significant fraction of MPs from WWTP, noting that the ceramic membrane was particularly effective at removing PE microplastics but less effective in rejecting PS microplastics and was less susceptible to productivity loss in filtering PE microplastics compared to polymeric membranes.

Sriani et al. [49] investigated the production of membranes from waste-expanded polystyrene (WEPS) containing up to 8% polyimide (PI). The membranes were tested for removal of MPs and bovine serum albumin. All membranes showed hydrophilic characteristics, good antifouling properties, and MP rejections above 80%. However, the one with 8% polyimide showed the lowest porosity and pore size values, while improving the antifouling property by 67% compared to the pristine membrane prepared solely with WEPS. The research spotlight was the investigation of waste with great potential for generating secondary MPs as raw material for producing membranes that could serve as a solution for removing MPs from aqueous matrices.

In fact, MF is the most studied PDMP for the removal of MPs, with Table 1 showing the conditions under which the MF studies evaluated in this work were conducted.

**Table 1:** Articles found in the literature related to microfiltration (MF) for the removal of microplastics (MPs).

Membrane						Operating Pressure (Bar)	Aqueous Matrix	Scale	Microplastic				Reference
Name	Supplier	Type	Material	Pore Size (µm)	Hydrophilicity				Type	Concentration	Size (µm)	Removal (%)	
VVLP	Millipore	Flat sheet	PVDF	0.10	Hydrophilic	0.7	Milli-Q water	Lab	PE	100 mg/L	<2	>90	[13]
									PVC	100 mg/L			
									PES	100 mg/L			
N.A. ^a^				≥0.2	N.A.	N.A.	RO water	Lab	PVC	39 ± 9 particles/L	39–246	93.6 ± 2.2	[23]
									PET	36 ± 7 particles/L	28–121		
									Nylon	64 ± 15 fibers/L	496–1862		
N.A.	Filter-Lab	Flat sheet	PC	5.0	Hydrophilic	0.5	Milli-Q water	Lab	PA	100 mg/L	20–300	~94	[45]
			CA		Hydrophilic				PS	100 mg/L	20–300		
			PTFE		Hydrophobic	1.5							
N.A.	Cembrane	Flat sheet	Silicon carbide	0.10	Hydrophilic	0.25	Laundry wastewater	Lab	PET	3000–45,000 fibers/L	29–36 thickness and 220–550 length	87.7–98.9	[46]
Meiden	Meindensha	Flat sheet	Al_2_O_3_	0.10	Hydrophilic	0.25	Laundry wastewater	Lab	PET	3000–45,000 fibers/L	29–36 thickness and 220–550 length	90.3–97.7	
T1-70, Membralox	Pall	Tubular	Al_2_O_3_	0.10	Hydrophilic	1.0	Synthetic wastewater (organic UV filter + water)	Lab	PE	100 mg/L	75–90; 300–355; 600–710	80.2–97.9	[47]
V0.1	Synder Filtration	Flat sheet	PVDF	0.10	Hydrophilic	1.0	DI water + surfactant	Lab	PE; PS	50 mg/L	0.1–1.0	>99	[48]
SteriLUX	Meissner	Flat sheet	PVDF	0.10	Hydrophilic								
Anopore/Anodisc	Whatman	Flat sheet	Al_2_O_3_	0.10	Hydrophilic								
N.A.	Lab-made	Flat sheet	WEPS/PI	0.17–0.20	Hydrophilic	1.0	DI water + SDS	Lab	PTFE	N.A.	7.0	>80	[49]
N.A.	Lab-made	Hollow fiber	Sericin coated on PP	N.A.	Hydrophilic	0.2	50 L DI water + 1.75 kg rock salt or sea salts	Lab	PE	287–417 particles/kg rock salt or 1434–2284 particles/kg sea salt	≥20–≤5000	99.30	[50]
									PET				
									PP				
									PVC				
									Nylon				
									PU				
									PS				
									Undefined				
N.A.	Pall ^b^	Hollow fiber	PVDF	0.10	N.A.	N.A.	Wastewater	Full	HDPE, LDPE, PP	N.A.	>5	>94	[51]
Durapore	Millipore	Flat sheet	PVDF	0.10	Hydrophilic	0.5	DI water + ethanol (1:1)	Lab	PE	50 mg/L	10–106	N.A.	[52]
				0.22									
				0.45					PA	50 mg/L	15–55		
				5.0									
N.A.	N.A.	Flat sheet	PVDF	0.22	Hydrophobic	1–3	Secondary wastewater	Lab	PE	200 particles/L	150	100	[53]
									PVC		250		
N.A.	Mervilab	Flat sheet	CA	5.0	Hydrophilic	0.1–0.7	Milli-Q water	Lab	PA	1–20 mg/L	10–105	N.A.	[54]
									PS		20–320		
N.A.	N.A.	Flat sheet	PES	0.22	N.A.	0.5	DI water	Lab	PE	10 mg/L	40–48	100	[55]
									PET	10 mg/L	300		
N.A.	ADVANTEC	Flat sheet	MCE	0.80	Hydrophilic	1.0	DI water	Lab	PS	2.5 mg/L	1.0	99.9	[56]
Filtanium	TAMI	Tubular	TiO_2_	0.80	N.A.	0.1–1.0	Secondary wastewater	Full	N.A.	10–100 particles/L	17–427	96	[57]
Filtanium	TAMI			0.14									
TÜBITAK 1-4	TÜBITAK			0.40									
N.A.	Lab-made	Flat sheet	Modified PVC	0.86–3.44	Hydrophilic	1.0	DI water	Lab	PS	2.5 mg/L	1.0	~100	[58]
N.A.	Lab-made	Flat sheet	Modified PVDF	0.34–1.36	Amphiphilic	1.0	DI water + 0.1% Triton X	Lab	PS	0.1%	0.1–0.5	90.41–97.2	[59]
N.A.	Lab-made	Flat sheet	Glass fiber membrane + chitosan-modified geopolymer sub-microparticles	0.058	Hydrophilic	0.9	Wastewater	Lab	PS	N.A.	0.05	92.04	[60]
N.A.	Lab-made	Flat sheet	SA/GO/CS	0.70	Hydrophilic	0.04	Synthetic wastewater	Lab	PS	N.A.	0.05	97.10 ± 1.17	[61]

Abbreviations: N.A.: not available; CA: cellulose acetate; CS: chitosan; DI: deionized; GO: graphene oxide; HDPE: high-density polyethylene; LDPE: low-density polyethylene; MCE: mixed cellulose ester; PA: polyamide; PC: polycarbonate; PE: polyethylene; PES: polyethersulfone; PET: polyethylene terephthalate; PI: polyimide; PP: polypropylene; PS: polystyrene; PTFE: polytetrafluoroethylene; PU: polyurethane; PVC: polyvinylchloride; PVDF: polyvinylidene chloride; SA: sodium alginate; SDS: sodium dodecyl sulfate; WEPS: waste-expanded polystyrene. ^a^ Point-of-use device composed of microfiltration + granular activated carbon + ion exchange resins. ^b^ from ref. [59].

### 3.2. Ultrafiltration

UF membranes have pores ranging from 1 to 100 nm and generally operate under pressures from 0.5 bar to a maximum of 10 bar. They have the capacity to remove suspended solids, colloids, and bacteria like microfiltration membranes, and can also be used to remove viruses and high-molecular-weight organic compounds from water and wastewater [43]. In addition of being a great alternative in the treatment of drinking water by removing algae, cyanobacteria, and other organic substances [62], UF can be used as pretreatment for reverse osmosis [63]. There are a variety of studies using UF, either individually or combining one or more processes, such as biological processes, activated carbon adsorption, electrocoagulation, and RO, among others, obtaining excellent results [64,65].

Ma et al. [16] conducted studies using coagulation and UF to remove MPs from water. Among their conclusions, they realized that coagulation alone was not sufficient to remove MPs from polymers such as PE, especially because its density is very similar to that of water. The removal efficiency was below 15%, showing some improvement with higher concentrations of Fe-based coagulant accompanied by an anionic polyacrylamide flocculant. The combination of coagulation with UF was also evaluated. Good performance was observed, especially in aspects related to fouling. When the flocs were smaller, a more compact cake on the membrane was formed, resulting in a greater drop in ultrafiltration flux. When larger MPs were present, larger flocs were formed and the cake became less compact, which resulted in higher permeate fluxes [16]. Pretreatment with Al-based coagulants exerts a better performance than those based on Fe [16,66]. In the presence of humic acid, the cake becomes thicker and even less compact due to the larger flocs, improving UF performance [16,66].

Furthermore, regarding the assessment of fouling on an UF membrane, during the processing of aqueous matrices intentionally contaminated with PET microfibers, Hachemi et al. [10] observed that the microfibers, in addition to only marginally influencing permeability, did not interact with the other components of the wastewater. The cake formed on the PVDF membrane showed similar compaction regardless of the presence or absence of PET microfibers. In other words, the presence of MPs did not significantly influence the system’s operation or its performance [10]. On the other hand, Ghasemi et al. [67] observed that the presence of MPs during the use of UF with hollow fiber membranes facilitated the removal of fouling caused by alginate. The membranes subjected to samples containing only alginate required a more thorough cleaning (hydraulic and chemical), whilst those subjected to samples containing alginate and MPs had less pore obstruction and the incrustation was released from the membrane with only hydraulic cleaning.

Yuan et al. [68] evaluated the performance of UF in two water treatment plants in Canada. The authors reported MP removals above 90%. Comparing different forms of filtration with various aqueous matrices, such as urban wastewater and wastewater, from a plastic recycling industry, González-Camejo et al. [69] highlighted the better performance of UF compared to gravitational filtration. However, differences were observed between the two operating modes regarding energy consumption, which in turn varied depending on the type of wastewater being treated [69]. This indicates the need to conduct pilot plant tests specifically for each type of aqueous matrix and desired objective. In addition, using pretreatments to reduce the MP load influent on the UF provides better membrane performance, mitigating the incidence of fouling. According to Xiong et al. [70], the presence of MPs favors the multiplication of bacteria inside the membrane; that is, they act as facilitators of biofouling.

In a study conducted in Thailand, UF achieved 96.97% efficiency in MP removal in a sewage treatment pilot plant [71]. Studies also underline the importance of MP removal methods that do not generate sludge, as the use of sludge from WWTPs or WTPs as a soil conditioner can be a major source of soil contamination due to the transfer of MPs from the liquid matrix (water or wastewater) to the solid matrix (sludge) [72].

Mitigating fouling is a crucial factor for the effective performance of membrane processes, which is why it has been the focus of many studies. In this regard, Enfrin et al. [12] performed UF tests with a commercial PSU membrane, observing significant fouling formation after 4 hours of ultrafiltration of samples containing micro- and nanoparticles. After 48 hours, the drop in flux throughout the tests became even more evident, along with the observation of an increasingly lower concentration of MPs in the retentate stream, confirming the suspicion that the MPs were adsorbed onto the membranes. Observations were also made using scanning electron microscopy (SEM) of the membrane before and after the tests, corroborating the findings regarding fouling. Focused on minimizing fouling issues, Enfrin et al. [73] conducted another study testing the intermittent injection of nitrogen gas into the feed stream during the UF of samples containing MPs and NPs to perform mechanical cleaning throughout the tests. Pristine PSU membranes and some modified with low-pressure plasma were tested. In all membranes, the gas injection together with the feed stream was able to reduce the adhesion of MPs on the membranes, resulting in better fluxes. Luogo et al. [74], aiming to remove micro- and nanoplastics from synthetic fabrics, tested two ceramic membranes: a silicon carbide (SiC) MF membrane and a zirconium oxide (ZrO_2_) UF membrane. The tests were conducted initially with synthetic wastewater containing nylon fibers and later with real wastewater from washing PVC tarpaulins. It was observed that the MF membrane displayed a drop in flux during the test with synthetic wastewater containing MPs, while the UF membrane remained constant. Both membranes showed complete rejection of MPs in both tests. However, in the test with real wastewater, the UF membrane showed a slight drop in flux throughout the experiment, but remained lower than the drop reached with the MF. In tests of 4 days of continuous operation, the MF membrane had a 95% decrease in permeate flux, while the UF exhibited only a 37% drop.

While some studies evaluated the modification of MF membranes for the removal of MPs, others focused on the modification of UF membranes, which demonstrated good anti-fouling performance, facilitating the cleaning and reuse of the membranes. This was observed by Fryczkowska and Przywara [75], who evaluated polyacrylonitrile (PAN) membranes with the addition of reduced graphene oxide. Mohana et al. [9] in turn prepared nanocomposite membranes based on a double-charged MOF (metal–organic framework), and the hydraulic permeability was superior to that of pristine membranes. In summary, UF has also been extensively studied for MP removal, as illustrated in Table 2, which outlines the main conditions under which the assessed studies were performed.

**Table 2:** Articles found in the literature related to ultrafiltration (UF) for the removal of microplastics (MPs).

Membrane						Operating Pressure (Bar)	Aqueous Matrix	Scale	Microplastic				Reference
Name	Supplier	Type	Material	Pore Size/MWCO	Hydrophilicity				Type	Concentration	Size (µm)	Removal (%)	
BY ^a^	Synder Filtration	Flat sheet	PVDF	100 kDa	Hydrophobic	0.5	DI water and synthetic wastewater	Lab	PET fibers	1 mg/L	0.142	>99	[10]
N.A.	Pureach Beijing	Flat sheet	PSU	30 kDa	Hydrophilic	1.0	Milli-Q water	Lab	PE (nano/microplastics from a facial scrub)	10 mg/L	0.013–0.69	N.A.	[12]
N.A.	Ande	Flat sheet	PVDF	100 kDa	N.A.	1.0	DI water + FeCl_3_·6H_2_O + 0.1 M NaHCO_3_	Lab	PE	N.A.	500–5000	100	[16]
N.A.	N.A.	N.A.	N.A.	N.A.	N.A.	N.A.	Landfill leachate	Full	PE, PES, PP, PA, EPM, PVAC	1.2 ± 0.57 particles/L	1000–5000 (41.7%); 500–1000 (16.6%); <500 (41.7%)	N.A.	[34]
N.A.	Yuling	Flat sheet	PES	100 kDa	Hydrophilic	0.8	DI water + humic acid (10 mg/L) + AlCl_3_·6H_2_O and FeCl_3_·6H_2_O	Lab	PS	100 mg/L	50	91.2–92.7	[66]
SIP-1013	Asahi Kasei	Hollow fiber	PSU	6 kDa	N.A.	0.11–0.15	DI water + alginate	Lab	PE	10–100 ppm	125	N.A.	[67]
(SIP-1023													
N.A.	Millipore	Flat sheet	PC	N.A.	N.A.	1.4	Drinking water	Lab	PET	>30 particles/L	74	>90	[68]
									PVC		61		
									PET		15		
									Nylon fibers		16		
KMS Puron	Koch	Hollow fiber	PVDF	0.03 µm	N.A.	0.05–0.45	Wastewater from a PET recycling plant and urban wastewater	Pilot	HDPE	5.8 ± 2.1 mg/L	25–500	100	[69]
									PET	12.3 ± 1.8 mg/L			
									Other MPs	0.17 ± 0.08 mg/L			
N.A.	Ande	Flat sheet	PVDF	100 kDa	Hydrophilic	0.05–0.7	Surface water from Qinghe River	Lab	PE	1 mg/L	40–48	N.A.	[70]
AAc	Lab-made	Flat sheet	PSU modified	N.A.	Hydrophilic	0.25–1.2	Milli-Q water	Lab	PE (nano/microplastics from a facial scrub)	10 mg/L	0.093	N.A.	[73]
CPAm					Hydrophilic								
HMDSO					Hydrophobic								
N.A.	LiqTech Ceramics	Tubular	ZrO_2_	0.074 µm	Hydrophilic	0.1–1.4	DI water	Pilot	Nylon fibers	180 mg/L	80	100	[74]
							Laundry wastewater		PVC fibers	~22.6 mg/L	N.A.	99.2	
N.A.	Lab-made	Flat sheet	rGO/PAN	0.15 µm	Hydrophilic	1–2	Wastewater from a PET recycling plant	Lab	PET	N.A.	N.A.	>80	[75]
N.A.	Motimo	Flat sheet	PVDF	100 kDa	N.A.	1.0	DI water + AlCl_3_·6H_2_O + 0.1 M NaHCO_3_	Lab	PE	N.A.	500–5000	100	[76]
UP150	Microdyn-Nadir ^b^	Flat sheet ^b^	PES ^b^	150 kDa ^b^	N.A.	1.0	Personal care product wastewater	Lab	N.A.	80 particles/L	N.A.	100	[77]

Abbreviations: N.A.: not available; DI: deionized; EPM: ethylene–propylene polymer; HDPE: high-density polyethylene; PAN: polyacrylonitrile; PC: polycarbonate; PE: polyethylene; PES: polyethersulfone; PET: polyethylene terephthalate; PP: polypropylene; PS: polystyrene; PSU: polysulfone; PVC: polyvinylchloride; PVDF: polyvinylidene chloride; rGO: reduced graphene oxide. ^a^ from ref. [78]. ^b^ from ref. [79].

### 3.3. Nanofiltration

NF is characterized by the usage of membranes with pores of 1 to 10 nm and operating conditions at pressures generally between 5 and 15 bar. In addition, it is capable of removing multivalent salts and organic molecules with molecular weights greater than 200 Da [43]. In many cases, NF is implemented as a polishing step after other treatments, usually aiming at water reuse.

In a study aimed at producing reused water from secondary effluent contaminated with microplastics, Lin et al. [80] assessed the influence of electrocoagulation as a pretreatment for nanofiltration. The combination of the two technologies improved the removal of MPs and reduced membrane fouling. However, the authors emphasized that attention must be paid to the applied electric current for electrocoagulation to be effective, ultimately contributing to better performance of the NF positioned downstream.

Akarsu et al. [77] also evaluated electrocoagulation followed by PDMPs for water reuse purposes, but for treating wastewater from personal care products containing microplastics. In electrocoagulation, various electrode materials, including iron, aluminum, stainless steel (SS), titanium, and graphite, were tested under different operating conditions. The effluent was subsequently treated with UF (UP150), NF (NF90 or NF270), or RO (BW30 or SW30) to produce reusable water. The SS electrode demonstrated the best performance in electrocoagulation. Under optimal conditions, it reduced the concentration of microplastics from 1030 particles/L to 80 particles/L and lowered chemical oxygen demand (COD) from 15,930 mg/L to 3200 mg/L (~80% removal). Although all PDMPs achieved complete removal of microplastics (see Table 3), only the SW30 RO membrane produced a permeate with a COD low enough for water reuse purposes.

When comparing the behavior of a polyamide NF membrane over 50 days of operation in treating two types of synthetic wastewater, Lin et al. [81] observed the influence of MPs on the incidence of fouling. The presence of MPs contributed to the formation of bacterial colonies and the accumulation of polysaccharides, proteins, and humic compounds in the NF membrane. It was also found that the wastewater containing MPs resulted in a significantly greater drop in permeate flux than that reached with the wastewater without MPs. However, the tested membrane performed well in rejecting MPs, achieving removal close to 100%. Furthermore, regarding the larger contaminants in the wastewater, such as polysaccharides, their rejection was not impaired, although there was greater fouling formation [81].

It is well known that landfills are also major sources of MP discharge and transport MPs and other contaminants through their leachate to watercourses [82]. When comparing the leachate treatment systems of two landfills in Istanbul, Kara, Sari Erkan and Onkal Engin [83] classified the MPs they found by size, color, and shape. Although the vast majority of MPs were fibrous in shape, which is already known from previous studies to hinder membrane performance, the UF and NF systems assessed achieved 96–99% MP removals, i.e., values much higher than those observed with conventional aerobic and anaerobic systems. In general, since most MPs were large, ranging in size from 0.5 to 2 mm, high removals were expected with the PDMPs assessed. Zhang et al. [34] investigated the distribution and removal characteristics of MPs in different stages of the leachate treatment plant of the Shanghai Laogang landfill (Shanghai, China), the largest in Asia. The leachate treatment plant uses pretreatment (homogenization), a biological system consisting of a membrane bioreactor (MBR) and a two-stage anoxic/oxic system, and advanced treatment (UF, NF, and RO). While the effluent from the plant (RO permeate) is discharged into the environment, the concentrates from the PDMPs and MBR are sent to the sludge treatment system and then disposed of in the landfill itself. According to the study, PE was the predominant type of MP found in the leachate, which correlated with the higher generation of waste from this material. Additionally, fibers represented the most abundant form, comprising 42.2% of the MPs. In terms of size, the largest particles were most common—58.3% measured between 0.5 and 5 mm—while the remaining 41.7% were smaller than 0.5 mm. Even though the leachate treatment plant removed a large portion, significant amounts of MPs were found in the NF and RO permeates. According to the authors, what may justify this finding is the possibility that the membrane itself releases MPs, since fractions of cellulose nitrate (CN) were found. Moreover, it is also possible that fibrous materials passed through the pores of the membranes due to their shape. Another important point mentioned by the authors is that the sludge formed during leachate treatment, as well as the concentrate from membrane processes, carried a high load of microplastics, which were returned to the landfill. Despite this practice being technically and environmentally appropriate, it does not definitively eliminate MPs, which remain in a cycle, being transferred from the solid phase (sludge or landfill soil) to the liquid phase (leachate), and are subject to migration into the environment through wind or leachate leaks.

Fortin et al. [84] focused their studies on evaluating MPs smaller than 20 µm and within the detection limit of Raman spectroscopy. Secondary post-treatment domestic sewage seeded with MPs was used as the aqueous matrix, which was then subjected to advanced treatments based on activated carbon and NF/RO followed by disinfection. The authors reported how difficult it was to work with MPs of those dimensions since contamination care needed to be redoubled throughout the process and Raman spectroscopy did not accurately detect particles smaller than 1 µm. Nevertheless, the treatments with NF and RO eliminated 100% of the MPs detectable by the analytical methodology. The main conditions under which the studies we found regarding the use of NF and RO for removing MPs are presented in Table 3.

**Table 3:** Articles found in the literature related to nanofiltration (NF) and reverse osmosis (RO) for the removal of microplastics (MPs).

Process	Membrane						Operating Pressure (Bar)	Aqueous Matrix	Scale	Microplastic				Reference
	Name	Supplier	Type	Material	MWCO	Hydrophilicity				Type	Concentration	Size (µm)	Removal (%)	
NF and RO	N.A.	N.A.	N.A.	N.A.	N.A.	N.A.	N.A.	Landfill leachate	Full	PE, PES, PP, PA, EPM, PVAC	1.2 ± 0.57 particles/L	1000–5000 (41.7%); 500–1000 (16.6%); <500 (41.7%)	N.A.	[34]
NF	NF270	Dow—FilmTec	Flat sheet	PA ^b^	400 Da ^a^	Hydrophilic ^a^	20	Personal care products wastewater	Lab	N.A.	80 particles/L	N.A.	100	[77]
	NF90				200 Da ^a^									
RO	SW30				99.4% NaCl rejection ^b^	Hydrophilic ^b^								
	BW30													
NF	NF90	Dow—FilmTec	Flat sheet	PA	200 Da ^a^	Hydrophilic ^a^	4.0	Synthetic wastewater	Lab	PET	1 mg/L	100	~100	[81]
NF	NF90	Dow—FilmTec	Flat sheet	PA	200 Da ^a^	Hydrophilic ^a^	4.0	Synthetic wastewater	Lab	PET	1 mg/L	100	100	[80]
RO	N.A.	N.A.	Spiral wound	N.A.	N.A.	N.A.	N.A.	Surface water	Full	Various types	0.96 ± 0.46 particles/L	20–5000	93 ± 5	[85]

Abbreviations: N.A.: not available; EPM: Ethylene-propylene polymer; PA: polyamide; PE: polyethylene; PES: polyethersulfone; PET: polyethylene terephthalate; PP: polypropylene. ^a^ from ref. [86]. ^b^ from ref. [87].

### 3.4. Reverse Osmosis

Reverse-osmosis membranes differ from other membranes due to their operating driving force. In addition to operating under high pressures (>20 bar), this means that transport through the membranes is based on the solution-diffusion mechanism. Their pores are smaller than 1 nm, and their applications range from obtaining high-purity water to the desalination of seawater, including for drinking water. Indeed, RO is one of the most important technologies for purifying water and wastewater [32,88]. Since it operates under higher-pressure conditions compared to other PDMPs, it incurs higher operating costs associated with its greater energy consumption [43]. Greater care must be taken when using RO to remove MPs, as the high pressure applied can fragment the MPs, reducing their size and generating nanoplastics [9]. This may be more relevant for plastics that have undergone weathering, which makes them more fragile and brittle.

Considering that many studies with PDMPs operating under milder pressures, such as MF, UF, and NF, have demonstrated success in removing MPs, few studies exist with RO for this purpose (see Table 3). Furthermore, thanks to RO mainly being used as a polishing step for other PDMPs, few studies have truly evaluated the influence of MPs on fouling formation in RO membranes, since MPs are generally removed in previous steps. Ziajahromi et al. [89] focused on developing sampling and quantification methods for MPs in wastewater and found MPs in different treatment stages of the evaluated WWTPs. In a complete WWTP, with primary, secondary, and tertiary treatment—including screening (3 mm mesh), sedimentation, biological treatment, flocculation, disinfection/dechlorination processes, UF, RO, and decarbonation—the authors found approximately 0.21 MPs per liter in the RO permeate. Considering the flow rate of the studied WWTP, this represents a discharge of 10 million MPs per day into the environment. The authors suggested the possibility of membrane imperfections, as well as internal sealing problems in the membrane modules or pipes [89]. Although a small fraction of MPs escaped from RO, it is worth noting that it is much more effective than conventional systems commonly used in WWTPs, making WWTPs sources of MPs released into water bodies [90]. The same can be said regarding the WTP tested by Dalmau-Soler et al. [85], in which advanced treatment methods, such as UF and RO, performed better for MP removal than conventional ones or those followed by ozonation and activated carbon. The authors emphasized that the complete treatment, including the stages of membrane processes, achieved a removal efficiency of 93% ± 5% of MPs, and evaluating the performance of RO alone, it managed to eliminate 54% ± 27% of the particles that remained from the other stages. Although microplastics cannot be completely eliminated, it is important to highlight the ability of RO to remove viruses, bacteria, and virtually all organic and inorganic contaminants, providing greater safety for the treatment of drinking water [85,91]. Another interesting contribution of Dalmau-Soler et al. [85] is that they investigated the migration of MPs from the hydraulic components of the WTP. They concluded that even with a low input, it was possible to find PVC fractions, for example, coming from the WTP’s own pipes. Therefore, the contamination of MPs in the system should be considered non-negligible.

RO membranes have also been modified and evaluated for MP removal. Farahbakhsh et al. [92] developed modified high-performance thin-film composite reverse-osmosis membranes, evaluating their hydraulic permeability, salt rejection, wettability, and fouling resistance, among other characteristics. The membrane that performed best in nanoplastic removal was the double-modified one, classified as TFC-RO-DM-2. This membrane was prepared using a PSU membrane as a base that underwent the incorporation of titanium-based metallic organic structures and multiwalled carbon nanotubes in the support layers and the selective layer, respectively. This modified bilayer membrane was able to remove NPs, achieving high permeate fluxes and good fouling resistance, attributed to its hydrophilic character and negative charge. The authors reported that it was possible to clean it with simple hydraulic washing.

As can be seen in Table 3, studies on NF and RO have predominantly focused on evaluating MPs in the micrometer and millimeter size ranges involving experiments performed on a laboratory scale or monitoring large-scale treatment plants. In the latter case, information regarding the membranes and operational conditions of the membrane processes or the treatment plants themselves is in fact scarce. It may seem counterintuitive to investigate NF and RO membranes, which have pore sizes below 10 nm, to remove particles as large as micrometer-scale. However, it is important to note that this may be related to the simple fact that larger particles are more easily detected and quantified than smaller ones, such as nanoplastics. Nonetheless, studies assessing large-scale treatment plants indicated the passage of MPs/NPs through both NF and RO membranes. While this may seem illogical, it could be attributed to a wider membrane pore-size distribution, imperfections in the membranes or modules, or even membrane aging. In addition, these studies did not report how long the membranes had been in operation, which is a critical factor, especially in the context of membrane application in leachate treatment, as was the case in the study by Zhang et al. [34]. In short, these are some hypotheses that may explain why NF and RO are being assessed to remove micrometric and even millimetric particles and also shed some light on the strange detection of MPs in NF and RO permeates.

## 4. Conclusions and Future Perspectives

This review presents a compilation of studies focused on PDMPs, unlike others that compared other strategies for removing MPs, allowing a comparison among the four types of PDMP. It was observed that most studies were carried out with MF or UF. This is associated with the fact that these PDMPs use membranes with smaller pores than the vast majority of MPs studied, which provides high removal rates. MF and UF operate at lower pressures than NF and RO and therefore have lower energy consumption, which is a crucial factor to consider in full-scale plants.

Among the studies analyzed, most were conducted on a laboratory scale using synthetic suspensions of microplastics in deionized or Milli-Q water or seeding them in some real (waste)water. In turn, some studies assessed industrial or domestic wastewater, landfill leachate, surface water, or water from different treatment stages in WTP or WWTP. Furthermore, studies performed with aqueous matrices consisting of synthetic wastewater or suspensions prepared with deionized or Milli-Q water were prepared with virgin polymer microplastics. Considering that the literature states that weathered MPs may present different behavior than virgin MPs, further studies evaluating PDMPs and other treatment systems in removing weathered MPs are necessary.

There was a predominance of studies whose concern in the preparation or functionalization of membranes or optimization of operational conditions focused on mitigating fouling and maintaining a stable permeate flux throughout the working regime. Indeed, new materials for manufacturing or modifying/functionalizing membranes to improve performance are a hot topic. Improvements in materials, preparation of 2D membranes, membranes based on graphene oxide, reduced graphene oxide, or MOFs, or membranes prepared with biodegradable materials can be applied in removing MPs, opening up a wide range of space for innovation in the field of material selection for membrane preparation.

Fouling is in fact the main drawback of membrane processes, which generally results in performance loss and increased operating costs. Although there are several studies on the role of MPs in membrane fouling, the information is still controversial, sometimes indicating that the presence of MPs in an aqueous matrix improves PDMP productivity or reporting the opposite; therefore, further studies are needed to consolidate the kinetics and mechanisms of fouling occurrence in different PDMPs.

Another important issue that requires in-depth investigation is defining the role of MPs in the clogging of feed channels in spiral-wound modules, particularly those of NF and RO, where spacer thickness typically ranges from 0.66 mm (26 mil) to 0.86 mm (34 mil) [93,94]. As shown in Table 1, Table 2 and Table 3, several studies have assessed the usage of PDMPs to remove MPs as large as 5 mm, demonstrating the potential for MPs to clog feed channels in spiral-wound modules containing NF or RO membranes. Similarly, despite having spiral-wound MF and UF modules featuring spacers thicker than 2 mm [78], their feed channels are also prone to clogging by MPs, especially by the larger ones, MP clusters, or MP agglomerates with colloidal substances.

Although some studies on rubber MPs exist, they evaluated treatment methods other than PDMPs. While microplastics are a recent subject of study and there are adversities related to the quantification and detection analyses of rubber microparticles, it would be interesting to evaluate the performance of PDMPs in their removal. This fact becomes even more important as the high deformation rates of rubbers, even under low shear stresses, can allow rubber MPs to undergo large deformations due to the operating conditions used in PDMPs, mainly due to the effect of pressure, and to pass through the membrane.

Considering the literature data we assessed, only one study cited a concern regarding the sludge generated from the PDMP concentrates and the pollutants adsorbed on the membranes. It is known that sludge treatments can fragment MPs during sludge drying by thermal processes or by lime stabilization. In addition, the sludge has more significant concentrations of MPs, often being disposed of in the soil, contaminating it to the point that it is possible to detect MPs in the soil even many years after sludge disposal. Therefore, studies based on the disposal of sludge and of PDMP concentrates rich in microplastics in the soil are necessary, aiming to investigate the interaction of these contaminants with different plant crops and soil microbiota. Physicochemical processes such as coagulation–flocculation–sedimentation (or flotation) could be an alternative for removing MPs from PDMP concentrates. In fact, the ideal would be to degrade or eliminate MPs from the environment, which in turn can be achieved by the action of microorganisms or worms, or even to evaluate the possibilities of recycling MPs or membranes, which would require an assessment of financial feasibility. Although chemical or mechanical recycling methodologies may not be feasible, avoiding the reintroduction of MPs into the environment is imperative.

Linking academic studies to the UN Sustainable Development Goals (SDGs) is an interesting way to demonstrate that the research is aligned with global goals and that the knowledge generated is relevant and has social and environmental impacts. In addition, associating a study with the SDGs can spark the interest of funding agencies, government agencies, or non-governmental organizations in making its implementation viable, which can drive public policies, economic transitions, and the provision of technologies to address crises such as climate change, for example. None of the studies evaluated in this review associated the removal of MPs with the SDGs, though some studies involving the removal of contaminants of emerging concern using membranes already made this connection. Goal 6—clean water and sanitation, in its subitems 6.3 and 6.a, and goal 14—life below water, in its subitems 14.1, 14.2, and 14.c are just two examples of SDGs with which a connection can be established with the removal of MPs by PDMPs. Considering the issues related to the management of PDMP concentrates and sludge formed in other treatment systems, there is also a relationship with SDGs 12—responsible consumption and production and 15—life on land, since PDMP concentrates and sludge generated in leachate treatment contain a high load of MPs, classifying them as causes of soil contamination. Both SDGs have targets that aim to reverse soil degradation and adopt sustainable waste management strategies to minimize negative impacts on human health and the environment. Considering that some areas of study related to water and wastewater treatment already use this association successfully, future work associating MP removal with SDGs tends to achieve promising results.

Indeed, MPs are emerging as a major concern, particularly for human health, but NPs may pose an even greater problem, since they can be as small as a few nanometers. This size allows for easier absorption into the human body, leading to deeper penetration into organs and tissues than MPs. Furthermore, MF and UF, the most commonly used PDMPs for MP removal, may be ineffective in removing small NPs. Additionally, some of the smallest NP particles could pass through NF membranes and also RO membranes. This is because the membranes have a pore size distribution, which means that they may have a fraction of pores larger and another of pores smaller than the nominal size, as well as some imperfections. On the other hand, despite concerns about micro- and nanoplastic pollution being a recent issue, several studies have shown that PDMPs are effective in removing these contaminants from aqueous systems. In most cases, rejections of almost 100% of particles were achieved, providing greater safety to the treatment system concerning the removal of these contaminants. Considering this and also the fact that PDMPs are capable of retaining contaminants of emerging concern whose removal in conventional treatment systems is ineffective, an expansion of the usage of membrane technologies in WTPs and WWTPs is expected in the near future, making it crucial to expand knowledge about the phenomena involved in the removal of MPs and NPs from water and wastewater by PDMPs.

## Data Availability

No new data were created or analyzed in this study. Data sharing is not applicable to this article.
